# Back to the Wild: The Parasitoid Community of *Lobesia botrana* (Lepidoptera: Tortricidae) in a Grapevine-Free Natural Environment

**DOI:** 10.3390/insects13070627

**Published:** 2022-07-14

**Authors:** Filippo Di Giovanni, Renato Ricciardi, Augusto Loni, Pier Luigi Scaramozzino, Giovanni Benelli, Andrea Lucchi

**Affiliations:** 1 Department of Agriculture, Food and Environment, University of Pisa, Via del Borghetto 80, 56124 Pisa, Italy; filippo.digiovanni@unisi.it (F.D.G.); renato.ricciardi@unipi.it (R.R.); augusto.loni@unipi.it (A.L.); pierscaramozzino@gmail.com (P.L.S.); andrea.lucchi@unipi.it (A.L.); 2 Department of Life Sciences, University of Siena, Via A. Moro 2, 53100 Siena, Italy

**Keywords:** biological control agents, *Campoplex*, European grapevine moth, hymenopteran parasitoids, Integrated Pest Management, *Trichogramma*

## Abstract

**Simple Summary:**

In a framework of sustainable agriculture, strategies aimed at preserving and enhancing pest natural enemies are crucial. However, knowledge about the parasitoid complex associated with a particular pest is often fragmentary. Herein, we investigated the parasitoids associated with the European grapevine moth, one of the main vine pests in the Mediterranean area, in a natural context, where the moth lives on *Daphne gnidium,* deemed as its original host plant. We observed a heterogeneous and complex community, consisting of a few predominant parasitoid species, followed by satellite species, and occasional parasitoids. Parasitic wasps, such as *Campoplex capitator* and *Trichogramma* spp., can be also found in the vineyards, thus understanding their dynamics in the wild could be useful to improve biological control strategies for managing EGVM populations.

**Abstract:**

The European grapevine moth (EGVM), *Lobesia botrana* (Lepidoptera: Tortricidae), is one of the major concerns for vineyard managers in the Mediterranean area. It is a polyphagous moth, which develops on a wide variety of host plants, among which the spurge flax, *Daphne gnidium* (Thymelaeaceae), very likely represents its originary wild host plant. In this study, we investigated the parasitoid complex of *L. botrana* feeding on *D. gnidium* during a three-year sampling in a natural reserve in Tuscany, Italy, where this plant is extremely abundant while the grapevine is absent. A total of 24 species of parasitoids were obtained from eggs, larvae, and pupae of EGVM, belonging to 6 families of Hymenoptera and a family of Diptera. The ichneumonid wasp *Campoplex capitator* was the most abundant larval parasitoid. Four species of the genus *Trichogramma* were obtained from parasitized eggs during the first year of sampling, with a peak in the parasitisation during the EGVM 3rd generation. Some of the main EGVM parasitoids on spurge flax were also observed in vineyards, although a certain degree of redundancy was observed in the wild, due to several less frequent “satellite” species exploiting the same host. Overall, this research sheds light on the parasitoid community and dynamics of this important moth pest in a grapevine-free natural ecosystem, discussing the possible role of natural areas as ecological reservoirs of pest natural enemies.

## 1. Introduction

Biodiversity conservation represents one of the main and attended objective of a sustainable agriculture [1,2]. Since the 1950s, when organochlorine insecticides were firstly introduced, concerns over side-effects of insecticides have grown, leading to reconsider insecticides as the lone control strategies against pests [3,4,5]. Consequences of insecticide overuse in agricultural settings include environmental pollution [6], toxicity towards non-target insects [7,8,9,10], and even risks for human health [11,12,13]. In this context, a strategy aimed at preserving pest natural enemies through environmental heterogeneity is encouraged. As predator and parasitoid insects rely on multiple resources for adult feeding and reproduction, enhancing the environmental heterogeneity can be a driver of their diversity at local scale, boosting species that forage on both semi-natural and crop habitats [14]. In addition, the presence of interconnected wild areas within an urban or rural landscape may promote species dispersal [15,16]. This concept applies well to the vineyard agroecosystem since the agricultural matrix can contribute to maintaining insect diversity by providing higher diversity of resources [17,18,19,20,21]. In Italy, the agricultural territory is characterised by a high fragmentation of the crops, with a landscape matrix where woods and Mediterranean scrubland habitats alternate with cultivated fields [14,22,23,24]. A good example is represented by the high-value vineyards characterizing Tuscany (Central Italy), a top producing wine region worldwide [4,14].

The European grapevine moth (EGVM), *Lobesia botrana* (Denis and Schiffermüller, 1775) (Lepidoptera: Tortricidae), is the main insect pest in most of the European wine-growing areas [25,26]. In recent years it has been introduced into the Americas, with first detections in Chile in 2008, California (USA) in 2009 and Argentina in 2010 [27,28]. EGVM is a polyphagous moth, which develops on a wide variety of host plants [29]. Among these, spurge flax, *Daphne gnidium* L. (Thymelaeaceae), very likely represents its originary wild host plant [30,31,32,33]. Several studies focused on the parasitoid complex of EGVM in the European vineyards [34,35,36,37,38,39,40,41,42,43,44,45,46,47,48,49,50,51,52,53,54,55,56,57,58,59], but very little is still known about parasitoids developing on wild EGVM populations [32,59,60,61,62,63,64]. In this scenario, we investigated the community structure of EGVM parasitoids on *D. gnidium* in a wild area where grapevine is absent, during a three-year sampling. Moreover, a comparison with the parasitoid species complex associated with EGVM in the vineyards is provided.

## 2. Materials and Methods

### 2.1. Study Area

**Figure 1:**
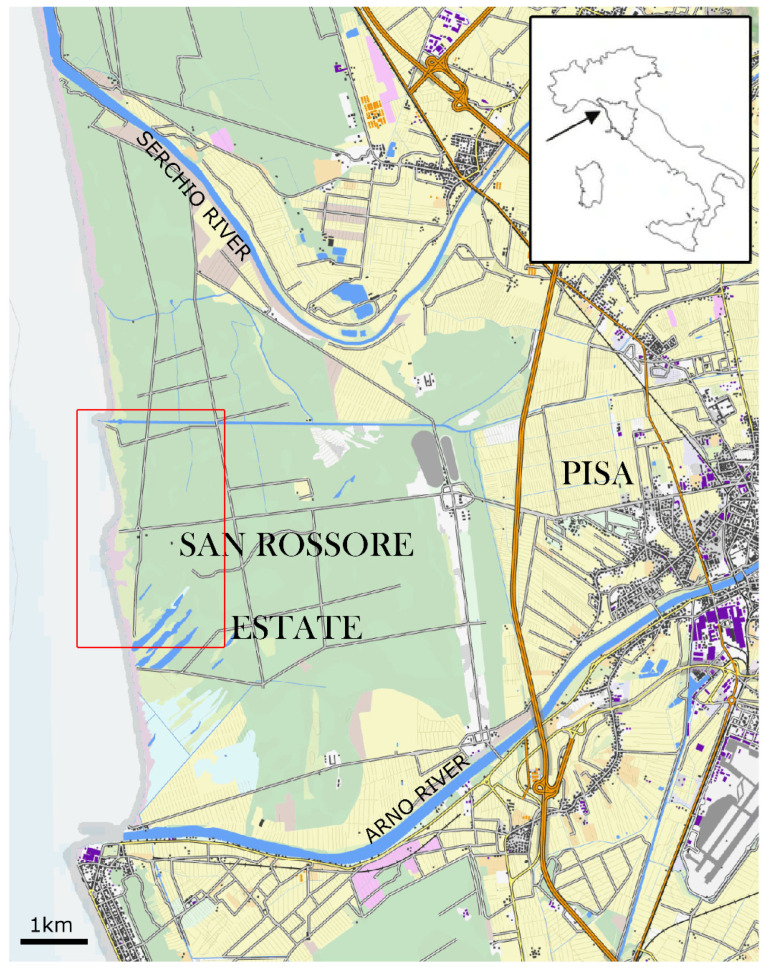
Map of the shoreline north of Pisa, showing the Regional Reserve of Migliarino, San Rossore, Massaciuccoli (Tuscany, Central Italy); the study area is delimitated in red.

The study was carried out in the Regional Reserve of Migliarino, San Rossore, Massaciuccoli (Tuscany, Central Italy), a natural area that extends for 23,100 ha along the seacoast, from Viareggio to Pisa (Figure 1). This reserve is characterized by a sub-humid climate [65], with a mixed holm oak wood (*Quercus ilex* L.), partly replaced by anthropogenic coastal pine forests on the ancient dunes. The forest is characterized by a prevalence of stone pine (*Pinus pinea* L.) followed by oaks, alders (*Alnus* spp.), ashes (*Fraxinus* spp.), and common oaks (*Quercus robur* L.), while the understory consists of common myrtle (*Myrtus communis* L.), lentisk (*Pistacea lentiscus* L.), narrow-leaved mock privet (*Phyllirea angustifolia* L.), and blackberry (*Rubus fruticosus* L.). Moving towards the seashore, the dune develops for about 200 m, and it is characterized by the maritime pine (*Pinus pinaster* Aiton), planted as a windbreak to protect the artificial inland stone pine plantation, followed by a severely depleted coastal portion with *Juniperus* spp. [66,67,68]. *D. gnidium* largely occurs in all these habitats [67,68], whereas the grapevine is absent, and the nearest commercial vineyards are about 6 km away. *D. gnidium* is particularly widespread in the open spaces, where the sunlight can easily penetrate. Starting from March to October, the sprouts, flowers, or fruits of the bushes can host a diverse community of lepidopteran preimaginal stages, while EGVM eggs and larvae represent a suitable and abundant resource for a rich community of predators and parasitoids [59,62,63,69,70].

### 2.2. Sampling Design

**Figure 2:**
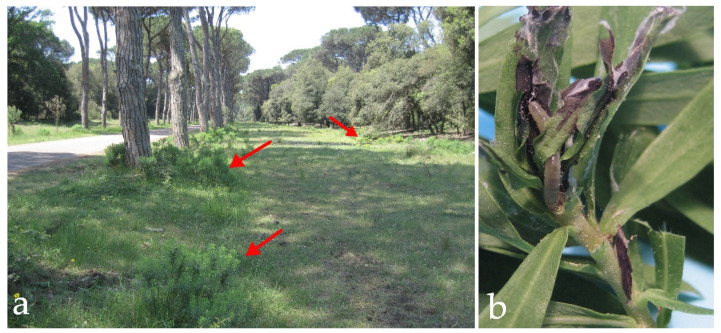
(**a**) *Daphne gnidium* shrubs (arrows) in the Regional Reserve of Migliarino, San Rossore, Massaciuccoli (Tuscany, Central Italy). (**b**) Nest of *Lobesia botrana* on a *D. gnidium* sprout.

Field observations were carried out inside the above-mentioned Natural Reserve in a selected rectangular-shaped area of about 150 ha, delimited by the following geographical points DD (43.733642° N, 10.277524° E; 43.712864° N, 10.279648° E; 43.732913° N, 10.292371° E; 43.720101° N, 10.293094° E). In this area, *D. gnidium* shows a spotted distribution, depending on the presence of open sunny spaces (Figure 2a). To get a homogeneous distribution of our samplings, a grid of 3 × 3 rectangles of 500 per 300 m side was superimposed on the selected area, being field observations on these nine sites carried out during 2014, 2015, and 2017. Each year, the sampling started at the end of April/beginning of May, with the appearance of the first visible nests of EGVM on *D. gnidium* sprouts, then lasting until the first week of October. Weekly, ten different plant per site were randomly selected and two infested sprouts for each plant were collected, totalizing 180 sprouts all over the selected area (Figure 2b). The sprouts were analysed under the stereomicroscope to isolate and identify EGVM preimaginal stages (1st instar larvae, 2nd–3rd instar larvae, 4th–5th instar larvae, and chrysalids). Preimaginal stages were stored in vials, supported with *D. gnidium* vegetal tissues as food, plugged with cotton to allow aeration, and maintained at natural environmental conditions of temperature and photoperiod until the insect emergence. Braconidae were identified by A. Loni and K. Samartsev (St. Petersburg, Russia) following Huddleston [71] and Loni et al. [62]; Chalcidoidea (excl. Trichogrammatidae) by Pier Luigi Scaramozzino, following Peck et al. [72]; Tachinidae by Filippo Di Giovanni, Pier Luigi Scaramozzino and Pierfilippo Cerretti (Rome, Italy) following Cerretti et al. [73]; Ichneumonidae have been identified by Filippo Di Giovanni and Pier Luigi Scaramozzino, following Aeschlimann [74], Kasparyan [75], Kasparyan [76], Gauld and Mitchell [77], Horstmann [78] and Tolkanitz [79]. In addition, from March to November 2015, the leaves and the tips of *D. gnidium* were examined to assess the presence of EGVM eggs parasitized by *Trichogramma* spp.; in each plot, the leaves of five buds on 20 shrubs were examined on a weekly basis, for a total of 100 shoots per plot. Eggs of EGVM were counted, distinguishing between healthy eggs and dark parasitized eggs. Leaves with parasitized eggs were stored in glass vials under controlled environmental conditions until parasitoid emergence. The emerged *Trichogramma* parasitoids were preserved in 96% (*v:v*) ethanol and identified at species level by Christoph Hoffmann (Siebeldingen, Germany) using the RFLP method and determination keys by Sumer et al. [80]; in one case (i.e., *T. cacaeciae* Marchal, 1927) ITS-2 DNA sequences were analysed at the Laboratory of Zoology and Integrated Production in Viticulture of the Julius Kühn-Institute (Quedlinburg, Germany) and compared to NCBI database. Results were partly published by Lucchi et al. [63]. All the insects were stored at the Department of Agriculture, Food and Environment, University of Pisa.

### 2.3. Host Parasitisation Rate and Statistical Analysis

For each year, the host parasitisation rate was calculated as the ratio between the number of emerged parasitoids and the total number of emerged specimens (hosts + parasitoids). The occupancy of the most represented species (i.e., species that have been collected in four out of nine of the selected sampling areas during at least one of the three years) was evaluated by considering the grid cells that contain at least one individual of the selected species on the nine sites of the grid [81,82]. Being *Campoplex capitator* Aubert, 1960 (Ichneumonidae, Campopleginae) the most abundant species of parasitoid emerged from infested sprouts of *D. gnidium*, the monthly emergence trend of this species during the three years was plotted and compared to that of EGVM using Kendall correlation coefficient (τ, significance threshold *p* < 0.05) as data were not normally distributed (test Shapiro-Wilk *p* < 0.05). The percentage of EGVM eggs parasitized by *Trichogramma* spp. in 2015 was counted, and a contingency analysis was carried out to test whether the number of parasitized eggs significantly varied over months. Finally, the community composition of parasitoids obtained from EGVM on *D. gnidium* was divided per guild, based on the attacked host stage. Then, parasitoids were further subdivided per guilds following Mills [83] and compared to the community composition of EGVM parasitoids as observed in vineyards or on spurge flax in different Italian regions [34,35,36,47,60,61,62,64].

## 3. Results

In our study, a total of 24 species of parasitoids were obtained from EGVM eggs, larvae, and pupae collected on *D. gnidium* during the three-year sampling in a grapevine-free natural environment (Table 1). From the total assemblage, 20 species of parasitoids belonging to 5 families of Hymenoptera (i.e., Braconidae, Eulophidae, Eupelmidae, Ichneumonidae, and Pteromalidae) and one family of Diptera (i.e., Tachinidae) were obtained from EGVM larvae and pupae collected during 2014, 2015, and 2017. In 2015, four species of parasitoids belonging to one family of Hymenoptera (i.e., Trichogrammatidae) were obtained from EGVM eggs collected on *D. gnidium* leaves. In 2014, 1223 adults of EGVM and 239 parasitoids were obtained, resulting in a host parasitisation rate of 16.35%. In 2015, 1070 adults of EGVM and 162 parasitoids were obtained, reaching a host parasitisation rate of 13.15%. During 2017, 1420 adults of EGVM and 163 parasitoids emerged, resulting in a host parasitisation rate of 10.30%. Overall, out of 3713 EGVM larvae or pupae, 564 parasitoids were obtained, with an average annual parasitisation rate of 13.27%. The family Ichneumonidae accounted for more than 80% of all the parasitoids, with a parasitisation rate ranging from about 9 to 12%. *C. capitator* was by far the most abundant species in our sampling, accounting for about 64% of all the emerged parasitoids, and with a parasitisation rate ranging from 7% to 9%.

**Figure 3:**
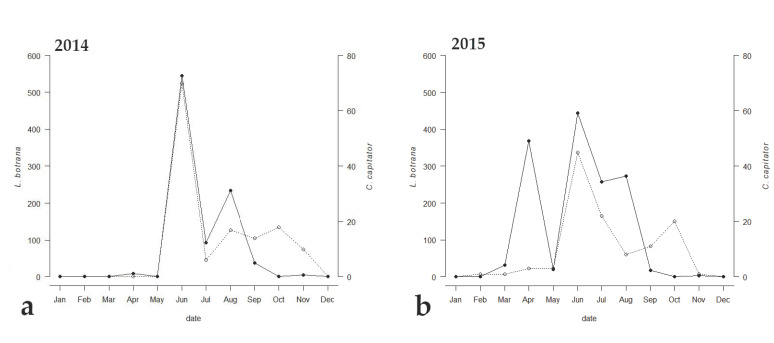

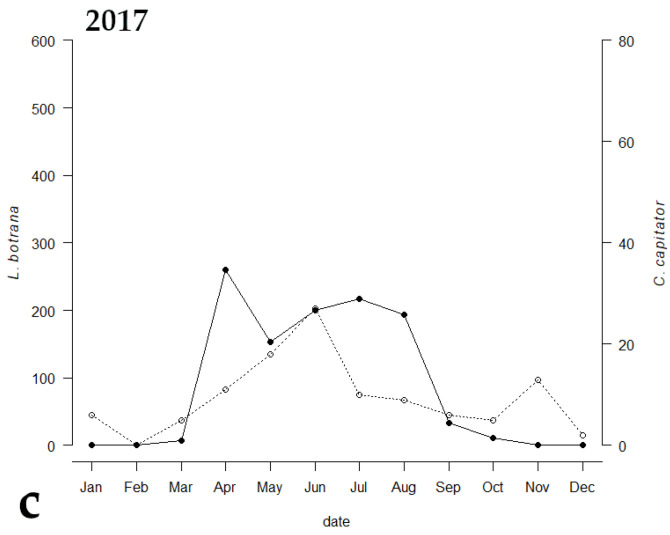
Emergence trends of *Lobesia botrana* (solid line) and its parasitoid, *Campoplex capitator* (dotted line), in (**a**) 2014, (**b**) 2015, and (**c**) 2017 in Tuscany, Central Italy.

Among the emerged parasitoids, only 8 species reached an occupancy threshold of at least 44%, (Table 2). Only two species (i.e., *C. capitator* and *Triclistus pallipes* Holmgren, 1873 (Ichneumonidae, Metopiinae)) reached an occupancy percentage value of at least 44% all the three years, with *C. capitator* having an occupancy of 100% during all the sampling periods. Comparing the emergence trend of *C. capitator* with that of EGVM, a strong correlation between the two curves was observed (Figure 3). The Kendall correlation coefficient was strong in 2014, when the trend of the graphs of the two species was almost identical (τ = 0.588, *p* = 0.0179), and less but still strong in 2015 and 2017 (τ = 0.484, *p* = 0.0355 and τ = 0.437, *p* = 0.0526 respectively).

**Figure 4:**
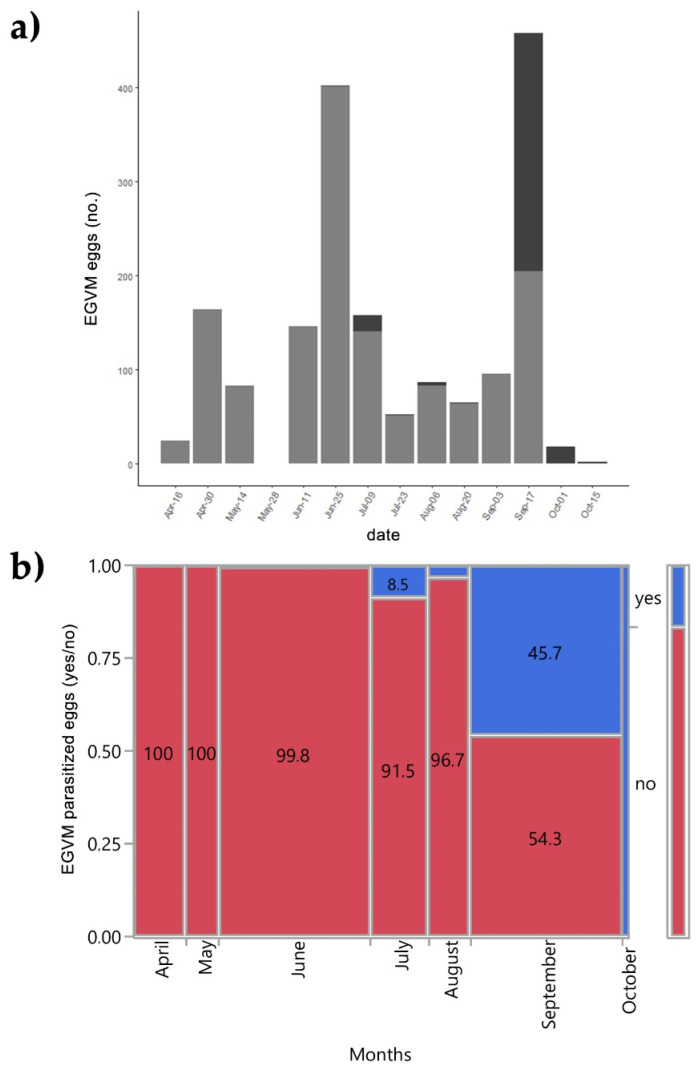
(**a**) Field parasitisation of *Lobesia botrana* eggs by *Trichogramma* spp. during 2015 in Tuscany, Italy; light grey: eggs of *L. botrana*; dark grey: eggs of *L. botrana* parasitized by *Trichogramma* spp. (**b**) Contingency analysis between *L. botrana* eggs parasitized by *Trichogramma* spp. and months during 2015. Values within tiles are percentages.

In 2015 the leaves and the tips of *D. gnidium* were also examined to assess the presence of EGVM eggs. Out of 1794 EGVM eggs found on the underside of leaves of *D. gnidium* shoots, 293 were parasitized by 4 *Trichogramma* species: *T. cordubense* Vargas and Cabello, 1985 (58.3%), *T. evanescens* Westwood, 1833 (35.4%), *T. euproctidis* (Girault, 1911) (4.2%) and *T. cacaeciae* Marchal, 1927 (2.1%) (see also Lucchi et al. [63]). The overall egg parasitisation rate was 16.33%. The number of parasitized eggs varies greatly over the months (χ^2^ = 665.42; *p* < 0.001). The first parasitized eggs were found at the end of June, during the second EGVM flight. No parasitized eggs were found between the end of August and the beginning of September. In mid-September, there was a strong resumption of *Trichogramma* spp. activity, with 271 out of 475 eggs parasitized, resulting in a parasitisation rate of 57% (Figure 4).

**Figure 5:**
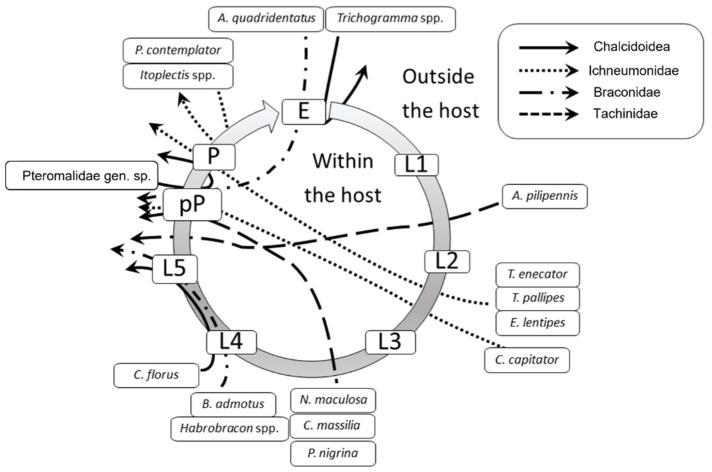
Parasitoids attacking different *Lobesia botrana* (EGVM) instars on *Daphne gnidium* sprouts in Tuscany, Central Italy. The grey circle represents the EGVM development. E: egg; L1-L5: I–V larval stages; pP: prepupa; P: pupa. Arrows connect the host stage attacked to the host stage killed by the parasitoid. Arrows passing through the host circle indicate an endoparasitic development, whereas those remaining outside the circle indicate an ectoparasitic development.

**Figure 6:**
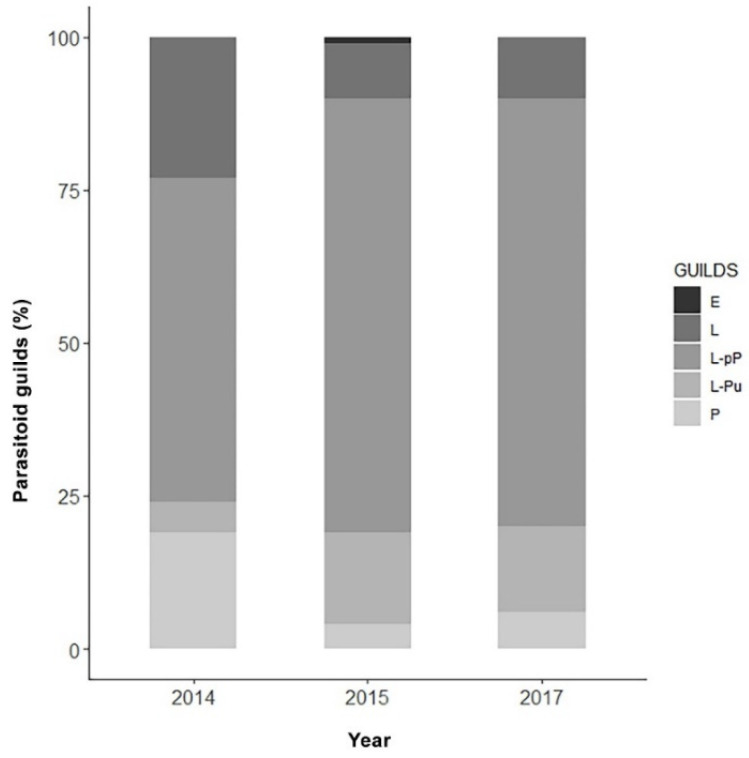
Parasitoid guilds (%) obtained across the three-year sampling in Tuscany, Central Italy, on the base of the host stage attacked. E: parasitoids attacking eggs (excluding *Trichogramma* spp.); L: larval parasitoids; L-pP: larvo-prepupal parasitoids; L-Pu: larvo-pupal parasitoids; P: pupal parasitoids.

Figure 5 shows the parasitoid community associated with EGVM on *D. gnidium* divided per guilds, based on the host stage attacked and the host stage killed by the parasitoid. In 2015, the analysis of parasitized eggs revealed that *Trichogramma* spp. and the braconid *Ascogaster quadridentata* Wesmael, 1835 were the only parasitoids attacking EGVM eggs, with the former completing development in the moth’s egg, and the latter emerging at the prepupal stage. Most of the parasitoids observed attack the early (such as *Actia pilipennis* (Fallén, 1810), *T. pallipes* or *C. capitator*) or late (such as *Habrobracon* spp. or *Colpoclypeus florus* (Walker, 1839)) larval stages of the moth, emerging from the last stages of host development. Observing the parasitoid community over the years, the larvo-prepupal guild (L-pP) was the relatively richest in terms of number of species, accounting for more than 50% in 2014, and about 70% in 2015 and 2017, followed by the larvo-pupal and pupal guilds (L-Pu and P) and by the larval parasitoids (L) (Figure 6).

## 4. Discussion

The list of parasitoids associated with EGVM is extremely long, and several authors have attempted to provide a complete list of species parasitizing this lepidopteran [84,85,86,87]. Not all these records should be considered valid, mainly due to inaccuracies in rearing, errors in identifications, and difficulties in interpreting names given by some authors in the past, as the taxonomic meaning of a name often changes over time or according to the author [69,70,88]. Most of the data comes from the investigation of the EGVM parasitoids in vineyards, as from the beginning this moth has been associated with the grapevine, showing its potential as a pest since the second half of 19th [69]. On the other hand, despite being considered the most important host plant of the EGVM and suggested as its original host [31,32], few Italian studies tried to highlight the population dynamics of this moth and its parasitoids on the spurge flax, *D. gnidium* [62,64]. In Italy, studies have been conducted in Tuscany [32,59,62,63,64], Apulia [60], and Sardinia [61], for a total of 28 (morpho)-species of parasitoids associated with EGVM developing on *D. gnidium* (Table 3). One of the major difficulties in identifying the correct host-parasitoid associations in a natural ecosystem is the coexistence of several lepidopteran species in the same microhabitat, which can be attacked in turn by unspecific parasitoids that exploit the habitat rather than the host [89]. An earlier investigation by Scaramozzino et al. [64] in the same area showed that the nests on sprouts of *D. gnidium* can be formed also by *Cacoecimorpha pronubana* (Hübner), a tortricid moth whose young larval stages can be confused with those of the EGVM. In addition, sprouts of *D. gnidium* often host a large community of moth larvae, belonging to several different species (e.g., leaf rollers such as *Anchinia cristalis* (Scopoli)), inquiline species such as *Cryptoblabes gnidiella* (Millère) or *Gymnoscelis rufifasciata* (Haworth), leaf-miners such as *Phyllobrostis fregenella* Hartig [64]. It follows that associating a parasitoid with its right host can be a difficult and time-consuming task [64,89].

The species diversity of parasitoids emerged from nests of EGVM on *D. gnidium* is slightly higher, although comparable, to that of the parasitoid community known from the few studies of EGVM parasitoids carried out in Tuscan vineyards [47], while the high prevalence of this plant in the Regional Reserve of Migliarino, San Rossore, Massaciuccoli made it possible to obtain much more information than in previous studies carried out on *D. gnidium* in Apulia and Sardinia [60,61]. Although a strict comparison between natural environment and vineyard is not possible, as a general trend, the parasitoid complex of EGVM in Tuscan vineyards is quite similar to what observed on *D. gnidium*, with many species in common between the two ecosystems, e.g., *C. capitator* Aubert, 1960 (Ichneumonidae), *T. pallipes* Holmgren, 1873 (Ichneumonidae), *A. quadridentata* Wesmael, 1835 (Braconidae), *Habrobracon pillerianae* Fisher, 1980 (Braconidae)*,* *T. evanescens* Westwood, 1833 (Trichogrammatidae) and *Phytomyptera nigrina* (Meigen, 1824) (Tachinidae). In addition, some sporadic species have been recorded both in vineyards and on *D. gnidium*, such as species of the genus *Exochus* (Ichneumonidae) or representatives of the genera *Itoplectis* (Ichneumonidae). Of note, few relevant species obtained in Tuscan vineyards [47] were not recorded in our study, i.e., *Elachertus affinis* Masi, 1911 (Eulophidae), *Dibrachys affinis* Masi 1907 (Pteromalidae) and *Dicaelotus inflexus* Thomson, 1891 (Ichneumonidae). This is probably due to an underestimation of the pupal parasitoid guild in our study; this component is usually sampled in vineyards, as the overwintering cocoons of EGVM can be easily found under the bark of vines or collected using rag-traps on vine stumps [42,44]. During the first two generations of the moth, the number of EGVM chrysalids on fronds of *D. gnidium* was quite low, while we miss to find overwintering pupae of the third generation on the plant, likely since the EGVM full-grown larvae leave the plant and pupate in the soil.

In our sampling, *C. capitator* resulted the most abundant parasitoid in all the three years, covering the 53%, 72% and 86% of the parasitoid community in 2014, 2015, and 2017, respectively. It was also the only parasitoid with a 100% occupancy in the selected area, proving to be a structural member of the community [81]. *C. capitator* is a Mediterranean species [87,88], widespread in most of the southern European wine-growing areas, where it is one of the main if not the most efficient parasitoid of EGVM [46,47,48,50,52,70,99,100]. Beyond EGVM, its host range seems restricted to a few other tortricid species feeding on grapevine, such as *Eupoecilia ambiguella* (Hübner, 1796) and occasionally *Sparganothis pilleriana* (Denis and Schiffermüller, 1775) [89]. Indeed, *C. capitator* belongs to a group of sibling species, which are difficult to separate on a morphological basis but that have probably differentiated based on host specialisation [88]. Our data strongly support the strict association of *C. capitator* with EGVM, as the trend of emergency of this parasitoid during the three-year sampling clearly followed that of the EGVM and gives credit to its possible use as a biological control agent against this pest as soon as the difficulties in its mass rearing can be overcome [101].

Apart from *C. capitator*, a plethora of “satellite species” appear in our sample, which although not reaching the parasitisation rates of the former, seem to be stably associated with EGVM. The ichneumonid *T. pallipes* Holmgren was the only species, together with *C. capitator*, to occur every year. This parasitoid species attacks the larval stages and emerges from the pupa and, although in relatively low numbers, showed a prevalent activity during the third generation of the moth. The tachinid fly *P. nigrina* (Meigen) has proven to be a constant presence, being found both on *D. gnidium* and in the vineyards in several studies [34,40,43,47,49,59,60,61,69]. This species seems to play an important role in the natural control of summer population of EGVM, sometimes reaching high parasitisation rates on 1st or 2nd generations of the moth [34,47,49,60,61]. However, the presence of this parasitoid is restricted to the Mediterranean area, so it can only be considered a suitable candidate for biological control of EGVM in southern European vineyards [52].

So far, 12 species of *Trichogramma* have been reported as parasitoids of EGVM eggs [98], some of which have been employed in inundative biological control against EGVM and *E. ambiguella* but with contrasting results [25,102]. Interestingly, *T. evanescens* was the only species of the genus collected in Italian vineyards [47]; the species is active during the 1st generation of the moth, when it can reach a parasitisation rate of about 25% [47]. In France, Hommay et al. [103] observed that *T. evanescens* is an efficient parasitoid of EGVM eggs during the 1st generation, while *T. cacoeciae* becomes more common during the 2nd generation. In Turkey, two species of *Trichogramma* were obtained from parasitized eggs of EGVM, with *T. euproctidis* being the most common one [56]. Interestingly, *T. cordubense* was by far the most abundant species of *Trichogramma* in our sampling to attack EGVM eggs on *D. gnidium*. In addition, this species did not attack the first generation of the moth, reaching the maximum parasitisation rate at the end of the third generation of the moth in September, when the parasitoid prolonged activity leads to the attack of all remaining laid eggs.

In general, what we observed in the wild is a complex community, with the impact of the different guilds that fluctuate over seasons and change over years, according to the different environmental conditions. Typically, the percentage of egg and larval parasitism is higher during the first two EGVM generations and decreases during the ovewintering generation, which is mainly affected by larvo-pupal and pupal parasitoids [25,47]. In our research, the larval endophagous guild exploited the great majority of EGVM larvae, mainly dragged by the high prevalence of *C. capitator*. However, when the parasitisation rate of this guild was lower (as in 2014), the incidence of the larval ectophagous and pupal parasitoids increased. This is consistent with the hypothesis that the members of these guilds would play a role as redundant species [104,105]. Therefore, this study highlights an extremely complex and dynamic parasitoid community; in this context, distinguishing parasitoid species that are closely associated with a host from occasional parasitoids and understand how this community varies across regions and seasons are two crucial aspects for the implementation of biological control strategies.

## 5. Conclusions

Eggs, larvae, and pupae of EGVM represent an abundant resource for a rich parasitoid community both in vineyards and in the wild. Despite a lack in sampling pupal parasitoids in our study, the community of parasitoids of EGVM on *D. gnidium* appeared highly diverse if compared to what observed in the vineyards. Some genera, as for *Trichogramma* or *Habrobracon*, were present with several species, showing off a certain degree of redundancy, with similar species exploiting the same resource and with one species that may replace another in the case of temporary or local extinctions [106]. In both ecosystems, *C. capitator* represents the main parasitoid of EGVM, confirming the importance of this species in the control of EGVM populations and its possible use as a biocontrol agent in vineyards [70,107,108]. Several other parasitoid species have been recorded in smaller numbers but appear to be stably associated with EGVM, suggesting that they may exert an important control on EGVM populations under local conditions.

This issue, coupled with the high number of EGVM parasitoid species in natural contexts, highlights the ecological value of wild areas as possible reservoirs from which even not common species with specific ecological requirements can move to reach surrounding cultivated areas, thus contributing to the control of economically important pests in the crops.

## Figures and Tables

**Table 1:** Overall abundance of *Lobesia botrana* and its larval/pupal parasitoids (in bold) on *Daphne gnidium* over three years of field studies; family names and total number of parasitoids per family are in bold; for each parasitoid family as well as for *Campoplex capitator,* the host parasitisation rate (%) is given within brackets.

Family and Species	2014	2015	2017	TOT
**Braconidae**	**30** (2.05)	**2** (0.16)	**5** (0.32)	**37** (0.87)
*Ascogaster quadridentata* Wesmael, 1835	-	1	-	1
*Bracon admotus* Papp, 2000	2	1	1	4
*Habrobracon concolorans* (Marshall, 1900)	9	-	-	9
*Habrobracon hebetor* (Say, 1836)	3	-	-	3
*Habrobracon pillerianae* Fisher, 1980	16	-	4	20
**Eulophidae**	**12** (0.82)	**12** (0.97)	**2** (0.13)	**26** (0.61)
*Colpoclypeus florus* (Walker, 1839)	12 *	12	2	26
**Eupelmidae**	**1** (0.07)	-	-	**1** (0.02)
*Eupelmus* sp.	1	-	-	1
**Ichneumonidae**	**181** (12.38)	**143** (11.61)	**145** (9.16)	**469** (10.97)
*Campoplex capitator* Aubert, 1960	128 (8.76)	114 (9.25)	112 (7.08)	354 (8.28)
*Exochus lentipes* Gravenhorst, 1829	1	3	-	4
*Itoplectis alternans* (Gravenhorst, 1829)	13 **	1	7	21
*Itoplectis maculator* (Fabricius, 1775)	3	-	-	3
*Itoplectis tunetana* (Schmiedeknecht, 1914)	25	4	3	32
*Pimpla contemplator* (Müller, 1776)	1	-	-	1
*Trichomma enecator* (Rossi, 1790)	7	-	-	7
*Triclistus pallipes* Holmgren, 1873	3	21	23	47
**Pteromalidae**	**3** (0.20)	**1** (0.08)	-	**4** (0.09)
gen. sp.	3	1	-	4
**Tachinidae**	**12** (0.82)	**4** (0.32)	**11** (0.69)	**27** (0.63)
*Actia pilipennis* (Fallén, 1810)	11	2	10	23
*Phytomyptera nigrina* (Meigen, 1824)	1	-	-	1
*Clemelis massilia* Herting, 1977	-	1	-	1
*Nemorilla maculosa* (Meigen, 1824)	-	1	1	2
**Total**	**239** (16.35)	**162** (13.15)	**163** (10.30)	**564** (13.19)
*Lobesia botrana* (Denis and Schiffermüller, 1775)	**1223**	**1070**	**1420**	**3713**

* *Colpoclypeus florus* is a gregarious parasitoid; the presence of more specimens from a single larva of *L. botrana* was counted as one event. ** 1 specimen obtained as hyperparasitoid of *Campoplex capitator*.

**Table 2:** Occupancy (%) for parasitoid species of *Lobesia botrana* that reached a value of at least 44% (i.e., 4 out of 9 sampled areas) during the three-year field study in Tuscany, Italy.

Parasitoid Species	Occupancy (%)		
	2014	2015	2017
*Actia pilipennis* (Fallén, 1810)	55	-	67
*Campoplex capitator* Aubert, 1960	100	100	100
*Colpoclypeus florus* (Walker, 1839)	55	44	-
*Habrobracon pillerianae* Fischer, 1980	-	-	44
*Itoplectis alternans* (Gravenhorst, 1829)	44	-	67
*Itoplectis tunetana* (Schmiedeknecht, 1914)	88	-	-
*Trichomma enecator* (Rossi, 1790)	44	-	-
*Triclistus pallipes* Holmgren, 1873	44	67	88

**Table 3:** Parasitoids of preimaginal stages of the European grapevine moth, *Lobesia botrana*, found on *Daphne gnidium* (D.g.) and *Vitis vinifera* (V.v.) in different Italian regions, based on main previous studies on the topic (references given in square brackets; black squares indicate presence). Species are divided into guilds as proposed by Mills [83]. Lifestyles: I, idiobiont; K, koinobiont; G, gregarious, H, hyperparasitoid; FH, facultative hyperparasitoid.

			Tuscany		Apulia		Sardinia	
	Host Plant	Lifestyle	*V.v.* [47,59]	*D.g.* [32,59,62,63,64]	*V.v.* [34,35,59]	*D.g.* [60]	*V.v.* [36]	*D.g.* [61]
Parasitoid Guild/Species [Ordo-Family]								
Egg endoparasitoid [EN]								
*Trichogramma cacaeciae* Marchal, 1927 [Hym-Trich]		I		■				
*Trichogramma cordubense* Vargas and Cabello, 1985 [Hym-Trich]		I		■				
*Trichogramma evanescens* Westwood, 1833 [Hym-Trich]		I	■	■				
*Trichogramma euproctidis* (Girault, 1911) [Hym-Trich]		I		■				
Egg-prepupal endoparasitoid [EpPN]								
*Ascogaster quadridentata* Wesmael, 1835 [Hym-Brac]		K	■					■
*Chelonus* sp. [Hym-Brac]		K					■	
Early larval endoparasitoid [ELN]								
*Phytomyptera nigrina* (Meigen, 1824) [Dipt-Tach]		K	■	■	■	■		■
Late larval endoparasitoid [LLN]								
*Actia pilipennis* (Fallén, 1810) [Dipt-Tach]		K		■				
*Neoplectops pomonellae* (Schnabl and Mokrzecki, 1903) [Dipt-Tach]		K		■				
Larval ectoparasitoid [LC]								
*Bracon admotus* Papp, 2000 [Hym-Brac]		I G?		■				
*Colpoclypeus florus* (Walker, 1839) [Hym-Eul]		KG		■				■
*Elachertus affinis* Masi, 1911 [Hym-Eul]		I G	■				■	
*Goniozus gallicola* (Kieffer, 1905) [Hym-Bet]		I [G (1)]	■					
Larval ectoparasitoid [LC]								
*Habrobracon* sp. [Hym-Brac]		I G					■	
*Habrobracon concolorans* (Marshall, 1900) [Hym-Brac]		I G		■				
*Habrobracon hebetor* (Say, 1936) [Hym-Brac]		I G		■	■			
*Habrobracon pillerianae* Fischer, 1980 [Hym-Brac] *		I G		■				
*Scambus elegans* (Woldstedt, 1877) [Hym-Ichn]		I FH					■	
Larval-prepupal endoparasitoid [LpPN] [a] Early attackers								
*Agathis* sp. [Hym-Brac]		K					■	
*Agathis malvacearum* Latreille, 1805 [Hym-Brac]		K			■			
*Aleiodes* sp. [Hym-Brac]		K				■		
*Apanteles* sp. [Hym-Brac]						■		
*Apanteles halidayi* Marshall, 1872 [Hym-Brac]		K			■			
*Campoplex borealis* (Zetterstedt, 1838) [Hym-Ichn]		K				■		
*Campoplex capitator* Aubert, 1960 [Hym-Ichn]		K	■	■				
*Campoplex difformis* (Gmelin, 1790) [Hym-Ichn]		K				■		
*Pristomerus vulnerator* (Panzer, 1799) [Hym-Ichn]		K					■	
*Therophilus linguarius* (Nees, 1812) [Hym-Brac]		K				■		
*Therophilus tumidulus* (Nees, 1812) [Hym-Brac]		K						■
Larval-prepupal endoparasitoid [LpPN] [b] Late attackers								
*Nemorilla maculosa* (2) (Meigen, 1824) [Dipt-Tach]		K		■				
*Clemelis massilia* (3) Herting, 1977 [Dipt-Tach]		K		■				
Larval-prepupal ectoparasitoid [LpPC]								
*Phytodietus* sp. [Hym-Ichn]		K	■					
*Phytodietus polyzonias* (Förster, 1771) [Hym-Ichn]		K						■
Larval-pupal endoparasitoid [LPN]								
*Exochus* sp. [Hym-Ichn]		K	■					
*Exochus lentipes* Gravenhorst, 1829 [Hym-Ichn]		K		■				
*Trichomma enecator* (Rossi, 1790) [Hym-Ichn]		K		■				
*Triclistus* sp. [Hym-Ichn]		K	■					■
*Triclistus lativentris* Thomson, 1887 [Hym-Ichn]		K					■	
*Triclistus pallipes* Holmgren, 1873 [Hym-Ichn]		K		■				
Prepupal-pupal ectoparasitoid [pPPC]								
*Agrothereutes pumilus* (Kriechbaumer, 1899) [Hym-Ichn]		I FH					■	
*Dibrachys affinis* Masi, 1907 [Hym-Pterom]		I FH	■		■		■	
*Dibrachys microgastri* (Bouche, 1834) [Hym-Pterom]		I FH					■	
*Ischnus alternator* (Gravenhorst, 1829) [Hym-Ichn]		I	■					
Pupal endoparasitoid [PN]								
*Dicaelotus inflexus* Thomson, 1891 [Hym-Ichn]		I	■				■	
*Hockeria* sp. [Hym-Chalc]		I	■					
*Itoplectis alternans* (Gravenhorst, 1829) [Hym-Ichn]		I FH		■			■	
*Itoplectis maculator* (Fabricius, 1775) [Hym-Ichn]		I FH	■					
*Itoplectis tunetana* (Schmiedeknecht, 1914) [Hym-Ichn]		I FH		■				
*Pimpla apricaria* Costa, 1885 [Hym-Ichn]		I					■	
*Pimpla spuria* Gravenhorst, 1829 [Hym-Ichn]		I	■					
*Pimpla turionellae* (Linnaeus, 1758) [Hym-Ichn]		I FH					■	
Hyperparasitoid								
*Bathythrix argentata* (4) (Gravenhorst, 1829) [Hym-Ichn]		H					■	
*Theroscopus hemipteron* (5) (Riche, 1791) [Hym-Ichn]		FH	■				■	
Uncertain placement								
*Pachyneuron* sp. [Hym-Pterom]		H			■			
*Tetrastichus* sp. (6) [Hym-Eul]		?			■			

* Several specimens obtained from EGVM 1st generation larvae on 13 June 2005 and from 2nd generation larvae on 1–20 August 2008 in Cerreto Guidi (Florence, Tuscany, Central Italy) (A. Lucchi leg.). (1) Larvae of *Goniozus gallicola* develop as solitary or gregarious parasitoids, depending on the size of the hosts [90]. (2) *Nemorilla maculosa* can also act as larval-pupal endoparasitoid [91]. (3) According to Scaramozzino et al. [59], this species can be assigned to the larval-prepupal endoparasitoid [LpPN] guild, but it is not known whether the passive penetration of the tachinid into the host larva occurs early or late. Rohdendorf [92] illustrated in detail the biology and development of another European species of the same genus, *Clemelis pullata* (Meigen, 1824), but the information provided does not allow to clarify this point. (4) This species has been recorded on EGVM by Catoni [93], Schwangart [94], Delrio et al. [36], Pinna et al. [38] and Marchesini and Dalla Montà [40]. Bordera and Selfa [95] report it from *Laelia coenosa* (Hübner, 1808) (Erebidae); its biology is poorly known, but according to Sawoniewicz [96] it develops as hyperparasitoid of lepidopteran larvae. (5) This species is reported as primary parasitoid of Lepidoptera (Noctuidae, Pyralidae, Tortricidae), Coleoptera (Chrysomelidae, Curculionidae) and Hymenoptera (Cephidae, Tenthredinidae) or as secondary parasitoid (hyperparasitoid) of Diptera Tachinidae through Hymenoptera Braconidae and Ichneumonidae [87]. Some authors consider it a true secondary parasitoid [97]. (6) The species belonging to the genus *Tetrastichus* Haliday, 1844 show very diversified lifestyles, associated with many hosts belonging to different orders of insects and arachnids [98]. Furthermore, many of the species once assigned to the genus *Tetrastichus* have been now assigned to different genera.

## Data Availability

The data presented in this study are available in article.
